# Yeast Stress, Aging, and Death

**DOI:** 10.1155/2013/684395

**Published:** 2013-12-02

**Authors:** Cristina Mazzoni, Sergio Giannattasio, Joris Winderickx, Paula Ludovico

**Affiliations:** ^1^Pasteur Institute-Cenci Bolognetti Foundation, Department of Biology and Biotechnology Charles Darwin Sapienza, University of Rome, 00185 Rome, Italy; ^2^National Research Council-Institute of Biomembranes and Bioenergetics, Via Amendola 165/a, 70126 Bari, Italy; ^3^Functional Biology, KU Leuven, Kasteelpark Arenberg 31, 3001 Heverlee, Belgium; ^4^Life and Health Sciences Research Institute, School of Health Sciences, University of Minho, 4710-057 Braga, Portugal; ^5^ICVS/3B's–PT Government Associate Laboratory, Braga, Guimarães, Portugal

Until about 15 years ago, programmed cell death (PCD), at that time mainly defined as apoptosis, was believed to be a feature occurring only in metazoans to ensure proper embryonic development, cell differentiation, and regulation of the immune response. However, the discovery that single-celled organisms, such as yeast, also undergo PCD challenged this idea. Meanwhile, several key regulators and cell death executers were shown to be highly conserved in yeast and other unicellular organisms, and it is now generally accepted that at least part of the molecular cell death machinery originated early in evolution.

Approximately 31% of the yeast genes have a mammalian homologue, and an additional 30% of yeast genes show domain similarity. This, combined with the ease of manipulation of yeast and the elegance of yeast genetics, has turned this lower eukaryote into an ideal system to study more complex phenomena that occur in metazoan cells, including stress responses, mechanisms governing life span and cell death, and processes contributing to aging or human diseases like cancers and neurodegenerative disorders.

In this special issue, we collected both original research and review articles, which combined give a nice overview on the current status of the field.

Several papers relate to mitochondrial functions and mitochondrial dynamics, thereby documenting the pivotal role of this organelle in aging processes and life span determination. In their review article, Y. Liu and X. J. Chen discuss a novel form of mitochondria-induced cell death in yeast cells tentatively referred to as degenerative cell death (DCD). Mutations in the adenine nucleotide translocase (Ant) cause aging-dependent DCD in yeast, which is sequentially manifested by inner membrane stress, mitochondrial DNA (mtDNA) loss, and progressive loss of cell viability. Recent work revealed that the Ant-induced DCD is suppressed by reduced cytosolic protein synthesis, suggesting a proteostatic crosstalk between mitochondria and the cytosol, which may play an important role in cell survival during aging.

Maintenance of mtDNA is important for cell growth and survival. Oxidative damage to mtDNA causes respiratory deficiency and human disease. In higher eukaryotes, the mechanisms for maintenance and transmission of the mitochondrial genome are still elusive. However, studies using the budding yeast *Saccharomyces cerevisiae *have generated an abundance of data on how its mitochondrial genome is maintained, and many nuclear-encoded proteins of diverse functions appear to be involved. As such, mutations in TCA cycle enzyme-encoding genes lead to variable defects in mtDNA maintenance and respiratory deficiency. The most severe phenotype is caused by mutations in the *ACO1 *gene encoding aconitase, which has been shown to have a novel function in mediating mtDNA maintenance by directly binding mtDNA. In this issue, Z. Liu and co-workers come with an alternative model to account for mtDNA loss due to an *aco1*Δ mutation. On the basis of genetic evidence they found that intracellular iron-citrate complex toxicity contributes to *aco1*Δ mutant phenotypes.

Mitochondrial morphology also changes during aging and similar changes are also observed when yeast cells switch their metabolism from the use of fermentative carbon sources to nonfermentative ones and vice versa. I. W. Dawes and co-workers studied this in more detail, which led them to a correlation between carbon source availability, mitochondrial dynamics, and autophagy. In particular, they show that increasing autophagy prevents mitochondrial fragmentation and that this relates directly to the determination of life span.

F. F. Severin and co-workers provide an overview and discussion on the role and delicate balance between mitochondrial fission and fusion events as an ingenious mechanism for the removal of defective mitochondria, a topic which directly links to the pathophysiological role of mitochondrial dynamics.

It is well known that genotoxic stress contributes to aging. Also, changes in the organization of chromatin are of particular importance. In their research paper, G. Miloshev and co-workers examined the role of the linker histone Hho1 on chromatin organization and found this histone to be essential for the maintenance of the chromatin loop structures during chronological aging.

Significant progress has also been made in understanding how metabolism impacts on aging and the determination of life span. In an interesting study, M. Barile and co-workers investigated the potential metabolic regulation of FAD by metabolites such as NAD, and they propose a novel role of mitochondrial NAD redox status in regulating FAD homeostasis in yeast.

M. J. Sousa and co-workers report that ammonium ion shortens the chronological life span (CLS) of *S. cerevisiae *cells when they are starved for different auxotrophy-complementing amino acids (leucine, lysine, and histidine). Interestingly, this effect of ammonium is mediated through different pathways depending on the amino acid that is missing. Therefore, this study provides interesting new insights on the underlying signalling network and as such gives new clues for the development of environmental interventions that extend CLS or for the identification of new therapeutic targets in diseases associated with hyperammonemia.

M. Vai and co-workers show that products of acetic acid and ethanol metabolism, rather than the compounds themselves, influence CLS. In particular, they show that inhibition of ethanol metabolism by pyrazole prevents CLS.

J. A. Bárcena and colleagues address two critical processes within the cell, that is, heme biosynthesis and the nonoxidative part of the pentose phosphate pathway (PPP). Particularly, the authors investigated the key enzymes uroporphyrinogen decarboxylase (Hem12p) and transketolase (Tkl1p) and proposed a redox control mechanism for heme biosynthesis that might be important in the context of tumour progression.

K. F. Cooper and colleagues reveal that Mtl1, a cell wall stress sensor protein that activates the cell wall integrity pathway (CWI) upon exposure to hydrogen peroxide, plays an important role in the pathways leading to destruction of cyclin C, which is involved in PCD.

S. Colombo and co-workers further investigated the involvement of the Ras proteins in PCD. They found that Ras-GFP relocalizes to mitochondria after abrogation of the glycolytic enzyme hexokinase 2. As this renders yeast cells more susceptible to acetic acid, their data suggest that this enzyme confers protection against apoptosis in *S. cerevisiae*.

R. Schaffrath and co-workers investigated how ceramides trigger stress responses in yeast. Besides being building blocks for complex sphingolipids in the plasma membrane, ceramides are known to play a crucial role in the regulation of cell proliferation and apoptosis. Here, the authors describe a novel Sit4-dependent regulatory mechanism in the ceramide stress response.

That metabolic changes play a crucial role not only at the cellular level, but also at the level of colonies is nicely documented in an elegant study by Z. Palkova and co-workers. Colonies represent a well-structured and differentiated multicellular community. During aging, cells within the colony produce ammonia as signal for metabolic reprogramming to support long term survival. Here, the authors reveal that aging giant colonies and rapidly developing microcolonies pass through similar developmental phases, which indicates that the age of colony is not crucial for colony differentiation.

Finally, three papers report how yeast can be used as a model to study human disease or to screen for drugs and decipher their mode of action.

V. Franssens and co-workers present an elegant review on the benefits of using humanized yeast models to study fundamental aspects related to protein folding diseases. The authors discuss the most important findings and recent advances that assign new roles for cell wall integrity signaling, Ca^2+^ homeostasis, mitophagy, and cytoskeleton-mediated transport processes in the pathobiology underlying Parkinson's disease.

The research article by K. Thevissen and colleagues demonstrates a role for superoxide dismutases in governing tolerance of the pathogenic yeast *Candida albicans* to the antifungal drug Amphotericin B.

The review article by G. Farrugia and R. Balzan illustrates how yeast research contributes to our understanding of the proapoptotic effects and the mode of action of traditional novel nonsteroidal anti-inflammatory drugs such as aspirin. The authors give an overview of the various proapoptotic pathways activated by these drugs and show the remarkable similarity of the effects triggered in yeast and mammalian cells.

In the opinion of the editors, the contributions published in this special issue highlight many novel features on the interplay between metabolism, stress responses, aging, and PCD pathways and how this determines the life span of yeast cells. Insight into the matter is not only important to better understand fundamental aspects of yeast physiology, but it is also of direct relevance in the context of human health, as nicely documented in several reviews.

